# Quantitative analysis of hemodynamic changes induced by the discrepancy between the sizes of the flow diverter and parent artery

**DOI:** 10.1038/s41598-024-61312-y

**Published:** 2024-05-09

**Authors:** Sunghan Kim, Hyeondong Yang, Je Hoon Oh, Yong Bae Kim

**Affiliations:** 1 Department of Neurosurgery, Bucheon St. Mary’s Hospital, College of Medicine, The Catholic University of Korea, Seoul, Republic of Korea; 2 Department of Mechanical Engineering and BK21 FOUR ERICA-ACE Center, Hanyang University, 55 Hanyangdaehak-ro, Sangnok-gu, Ansan, Gyeonggi-do 15588 Republic of Korea; 3 Department of Neurosurgery, Severance Hospital, Yonsei University College of Medicine, 50-1 Yonsei-ro, Seodaemun-gu, Seoul, 03722 Republic of Korea

**Keywords:** Flow diverter, Cerebral aneurysm, Metal coverage rate, Computational fluid dynamics, Hemodynamic changes, Size discrepancy, Neurology, Neurological disorders

## Abstract

The efficacy of flow diverters is influenced by the strut configuration changes resulting from size discrepancies between the stent and the parent artery. This study aimed to quantitatively analyze the impact of size discrepancies between flow diverters and parent arteries on the flow diversion effects, using computational fluid dynamics. Four silicone models with varying parent artery sizes were developed. Real flow diverters were deployed in these models to assess stent configurations at the aneurysm neck. Virtual stents were generated based on these configurations for computational fluid dynamics analysis. The changes in the reduction rate of the hemodynamic parameters were quantified to evaluate the flow diversion effect. Implanting 4.0 mm flow diverters in aneurysm models with parent artery diameters of 3.0–4.5 mm, in 0.5 mm increments, revealed that a shift from oversized to undersized flow diverters led to an increase in the reduction rates of hemodynamic parameter, accompanied by enhanced metal coverage rate and pore density. However, the flow diversion effect observed transitioning from oversizing to matching was less pronounced when moving from matching to undersizing. This emphasizes the importance of proper sizing of flow diverters, considering the benefits of undersizing and not to exceed the threshold of advantages.

## Introduction

Flow diverters (FDs) have changed the approach to treating large and giant intracranial aneurysms, and they represent a crucial component of modern aneurysm management. These devices work by altering the hemodynamic environment within the aneurysm to promote thrombogenesis, leading to occlusion of the aneurysmal sac^1,2^. The effectiveness of an FD, represented by the flow diversion effect, is significantly governed by the metal coverage rate (MCR), which is a measure of the proportion of the aneurysmal neck covered by the stent after implantation^3^. The MCR is influenced by the discrepancy between the sizes of the FD and parent artery^4,5^. Therefore, selecting an appropriate FD size relative to the parent artery diameter has significant clinical implications for the successful treatment of large and giant intracranial aneurysms.

Meanwhile, several studies have investigated the effect of MCR changes due to size discrepancies between FD and parent arteries on flow diversion effects. These investigations have enhanced our understanding of the impacts of oversizing^6^, or undersizing^7^ and compaction^8^ of the FD on the flow diversion effect, facilitating practical application in clinical settings. However, beyond the general recognition of undersizing or oversizing impacts, a precise quantitative analysis is crucial to delineate the relationship between specific size discrepancies of FDs and parent arteries and their effect on flow diversion. This detailed correlation is vital for establishing a more effective sizing strategy for FDs. Therefore, the quantitative impact of FD and the parent artery size discrepancies on aneurysm flow dynamics and treatment outcomes require further investigation. The purpose of this study is to quantitatively compare and analyze the changes in the flow diversion effect caused by the discrepancy between the sizes of an FD and the parent artery using a commercially available FD and computational fluid dynamics analysis. We aimed to determine whether undersizing the FD could significantly improve the flow diversion performance to contribute valuable insights to optimize clinical decisions and enhance treatment outcomes for patients with cerebral aneurysms.

## Methods

Uniform-diameter FDs were implanted in idealized aneurysm models with various parent artery sizes to conduct a quantitative analysis of the flow diversion effect of the discrepancies in the sizes of the parent artery and FD. The arrangement of wires in the FD installed in each aneurysm model was thoroughly examined to generate a virtual stent configuration. Finally, comparative flow analysis with computational fluid dynamics was performed using a virtual aneurysm of the same specification as the idealized aneurysm model and a previously generated virtual stent configuration. The individual methodologies for each step are outlined below.

### Aneurysm and flow diverter modeling

An idealized sidewall-type saccular aneurysm model was initially developed. The model was assumed to have a radius of 5.0 mm, representing a large aneurysm. To examine the effects of variations in the flow inside the aneurysm, the diameters of the parent artery were modified to 3.0 mm, 3.5 mm, 4.0 mm, 4.5 mm, and 5.0 mm. The manipulation of the neck diameters ensured a consistent neck area for comparative analysis, irrespective of changes in the parent vessel diameter.

**Figure 1:**
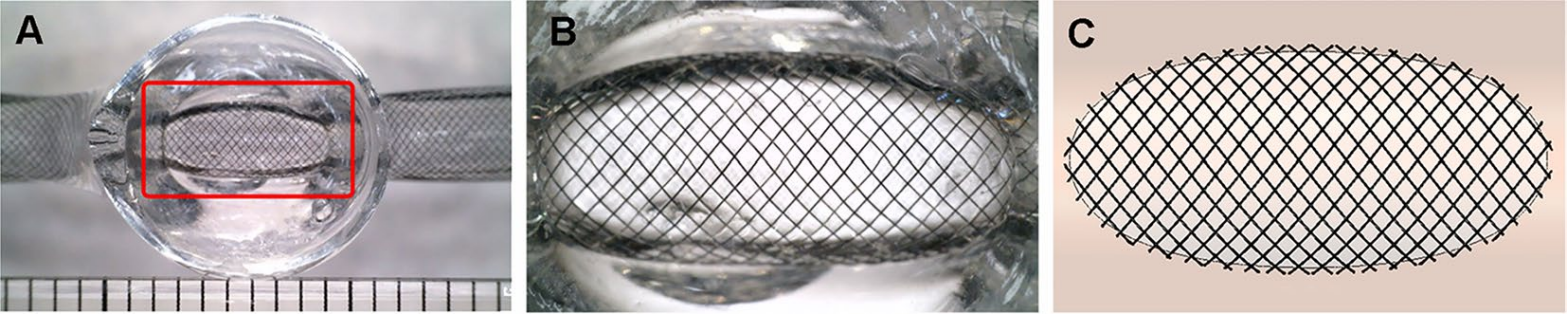
Steps to generate virtual stent for computational fluid dynamics analysis. (**A**) An artificial vascular replica of the idealized sidewall-type saccular aneurysm model with a real pipeline embolization device (PED) installed (indicated by a red square). (**B**) A detailed view of the PED deployed within the silicone aneurysm model. (**C**) Virtual stent created based on the wire configurations of the real PED.

The Pipeline Embolization Device (PED; Medtronic Neurovascular, Irvine, CA, USA) with a diameter of 4.0 mm was employed as an FD. The optimal length of the PED was determined based on an evaluation of the degrees of compaction in the parent vessels, which had varying diameters. The artificial vascular replicas of the idealized aneurysm model were fabricated using the liquid-assisted dip coating method, enabling the acquisition of the geometries of each PED (Fig. 1A)^9^. Subsequently, each flow diverter was delivered and deployed using the Marksman microcatheter (Medtronic Neurovascular). In deploying the PED, we consistently employed the least amount of device manipulation required to achieve proper flow diverter apposition with the parent vessel wall to prevent deliberate compaction at the aneurysm neck. The reliability of the measured MCR was verified by referring to the reference values from previous studies^10^. The geometries of the deployed PEDs were captured through imaging with a Micro-USB camera (AM7115MZT Dino-Lite Edge, Dino-Lite) (Fig. 1B).

To generate computational models of the flow diverters, the obtained geometries from the Micro-USB camera were referenced, and a commercial computer-aided design program (CATIA, V5-6R2012; Dassault Systèmes, Paris, France) was employed. The PED wires were exclusively constructed at the aneurysm neck to improve the efficiency of the computational fluid dynamics (Fig. 1C)^11^. The metal coverage rate (MCR, the percentage of the aneurysmal neck covered by metal after the application of a stent)^12^ and pore density (the number of pores per unit surface area)^11^ were calculated based on the geometries of PEDs. Figure 1 shows the geometries of the deployed PEDs within the artificial vascular replica and the corresponding computational flow diverter models.

### Validation of the computational fluid dynamics

**Figure 2:**
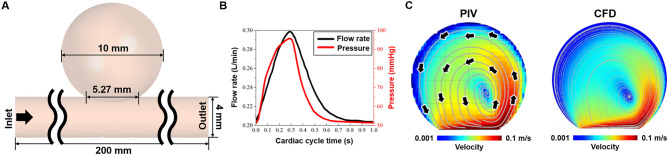
Comparison of computational fluid dynamics (CFD) and particle image velocimetry (PIV) results. (**A**) Idealized sidewall-type saccular aneurysm model. (**B**) Pulsatile flow rate and pressure profiles of CFD boundary conditions. (**C**) Velocity contours inside the aneurysms obtained from CFD and PIV. The velocity contour and streamline computed by CFD show strong consistency with the PIV experiment, indicating that the CFD employed in this study accurately captures the actual blood flow dynamics within a cerebral aneurysm.

Computational fluid dynamics was employed to evaluate the changes in flow inside the aneurysms in relation to the variations in the parent vessel diameters. Before investigating the flow using the idealized sidewall-type saccular aneurysm model (Fig. 2A), a validation process was conducted to ensure the reliability of the computational fluid dynamics. During this validation, we utilized experimental data from Tupin et al.^13^, who performed a particle image velocimetry (PIV) experiment on the idealized sidewall-type saccular aneurysm.

An element size of 0.2 mm was used for the validation model, while the density and viscosity of fluid were set to 1200 kg/m^3^ and 0.0038 Pa·s, respectively. The inlet and outlet boundary conditions during their experiment were incorporated into our computational fluid dynamics validation process to facilitate the comparability of our findings with those of Tupin et al. (Fig. 2B). Velocity profiles and streamlines were extracted after three cardiac cycles. Meshing and computational fluid dynamics simulations were carried out using the ANSYS Workbench Fluent (version 2019R1; ANSYS Inc., Canonsburg, PA, USA). The velocity contour and streamline calculated through computational fluid dynamics showed consistency with the outcomes of the PIV experiment (Fig. 2C).

### Computational fluid dynamics analysis with stent

To investigate the influence of flow diverters on flows inside the aneurysm, we referred to the computational fluid dynamics methodology previously developed by Kim et al.^14^. Based on the mesh convergence test, an element size of 0.2 mm was utilized for the aneurysm, while a finer element size of 0.005 mm was used in the flow diverter deployment site (Supplementary Figure S1). Overall, 30–50 million elements were used for computational fluid dynamics. The blood was treated as an incompressible Newtonian fluid, characterized by a density of 1,055 kg/m^3^ and a viscosity of 0.004 Pa·s^15^. The pulsatile flow profile of the internal carotid artery, incorporating the Womersley profile, was employed as the inlet boundary condition, while a zero pressure was implemented at the outlet boundary condition^16^. The blood vessel was considered as a rigid wall to adhere to nonslip conditions. All hemodynamic parameters were calculated during the systolic phase after three cardiac cycles^17^.

For a quantitative evaluation of the computational fluid dynamics, we compared several hemodynamic parameters, including the inflow rate, average velocity, and energy loss^18,19^. The inflow is defined as the normal velocity component of the aneurysm neck plane. The energy loss refers to the quantity of blood entering the aneurysm by evaluating the difference between the energies in the proximal and distal sites of the aneurysm. To measure the efficacy of flow diverters, we computed the reduction rate of the inflow, average velocity, and energy loss. Higher reduction rates of these parameters indicated lesser blood flow into the aneurysm. In addition, the wall shear stress (WSS) at systole, time-averaged WSS (TAWSS), and oscillatory shear index (OSI), which have a high correlation with aneurysm formation, growth, and rupture, were evaluated^20^.

## Results

### Comparison of metal coverage rate depending on the discrepancy between the sizes of the parent artery and flow diverter

**Figure 3:**
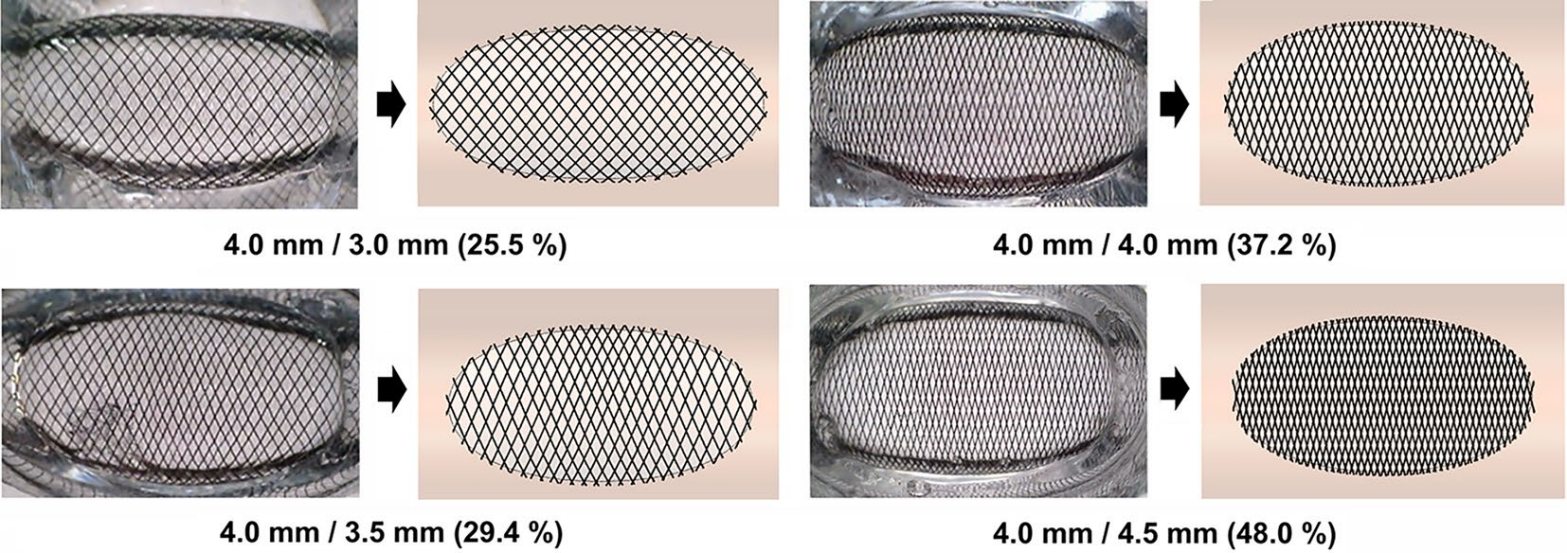
Configurations of wires of the pipeline embolization device (PED) deployed in aneurysm models and their computational counterparts, reflecting size discrepancies between the diverter and the parent artery. The metal coverage rate (MCR) increased from 25.5 to 48.0% as the size discrepancy changed from the 1.0 mm oversizing to 0.5 mm undersizing. The numbers beneath each image indicate the diameter of the flow diverter (FD), diameter of the parent artery, and MCR, respectively.

Figure 3 shows the arrangement of stent struts in the PEDs with a 4.0 mm diameter when installed in four distinct ideal aneurysm models with parent arterial diameters of 3.0 mm, 3.5 mm, 4.0 mm, and 4.5 mm, respectively. The installation of the PED in a 5.0 mm parent artery was excluded from the experiment due to inadequate wall apposition even after applying maximum compaction of the stent. Visual inspection of the stent struts showed a higher degree of compaction when the PED was implanted in an aneurysm model with a parent artery size of 4.5 mm than that with a parent artery size of 3 mm.

To assess the MCR based on the discrepancy between the sizes of the stent and the parent artery, the configurations of each implanted PED in the aneurysm model were captured, and computational models were constructed and subjected to comparative analysis. The MCR of the PED implanted in the aneurysm model with parent artery diameters of 3.0 mm (1.0 mm oversizing), 3.5 mm (0.5 mm oversizing), 4.0 mm (size matching), and 4.5 mm (0.5 mm undersizing) were 25.5%, 29.4%, 37.2%, and 48.0%, respectively. This demonstrates the propensities of both the compaction rate of stent struts and the MCR to increase with the transition from oversizing to undersizing.

### Comparison of the flow diversion effect depending on the discrepancy between the sizes of the parent artery and flow diverter

**Figure 4:**
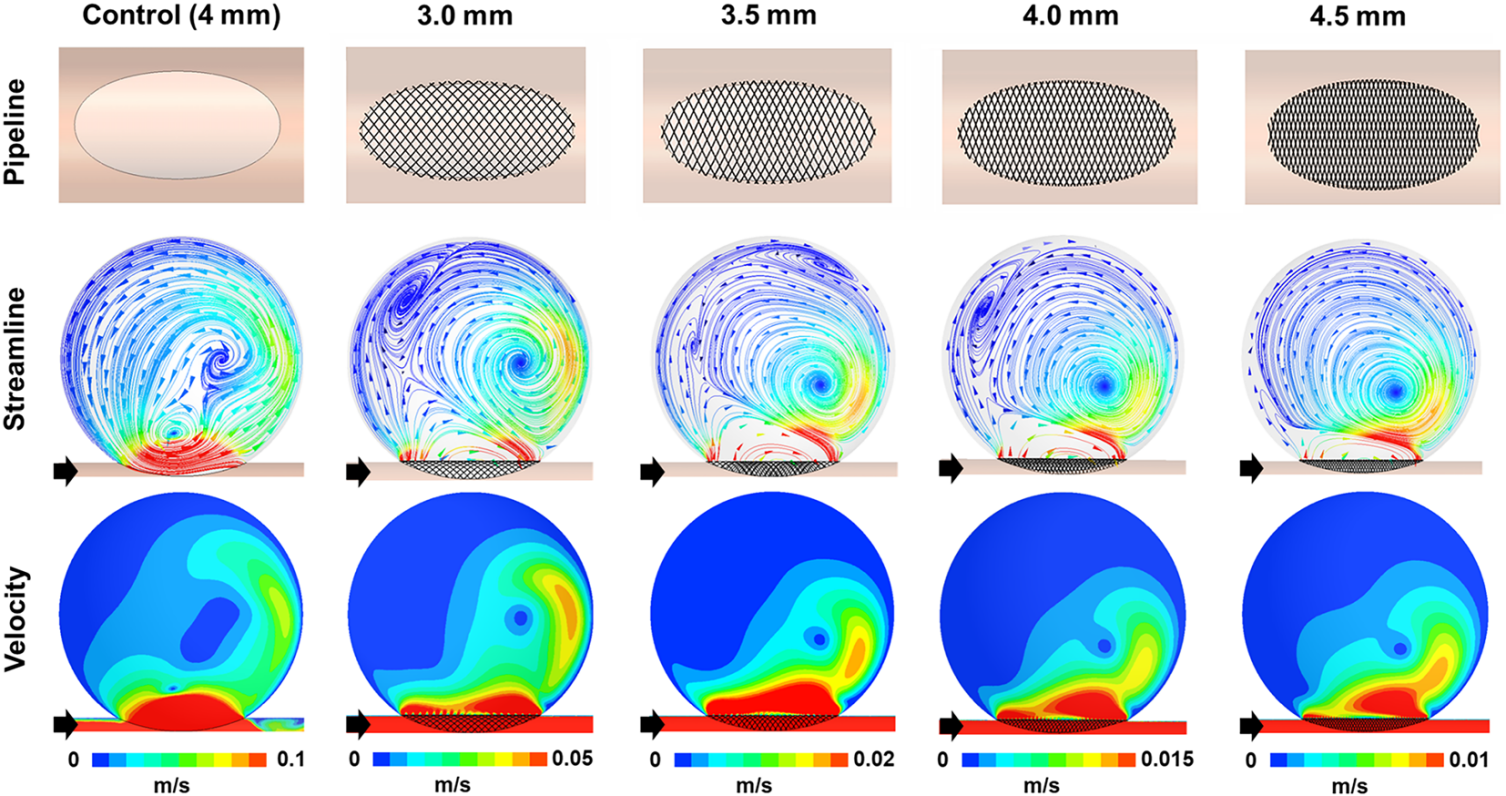
Impact of the discrepancy between the sizes of the flow diverter and parent artery on the flow diversion effect. The first row represents the configurations of a 4.0 mm pipeline deployed in the idealized aneurysm model; each was tailored to the size of the parent artery. The second and third rows illustrate the streamlines and velocity contours determined using computational fluid dynamics analysis for both before and after the deployment of the pipeline embolization device (PED). The direction of the flow is indicated by black arrows. Regardless of the size of the parent artery, the aneurysm model with the PED deployed showed intra-aneurysmal flow diversion. However, a higher metal coverage rate (MCR) resulting from size matching or undersizing led to a decrease in the volume and velocity of the inflow jet. This indicates an enhanced flow diversion effect of undersizing compared to oversizing.

Figure 4 shows the results of the computational fluid dynamics analysis, highlighting the influence of the discrepancy between the sizes of the PED and the parent artery on the flow diversion effect. Irrespective of the size of the parent artery, all aneurysm models in which PEDs were deployed showed intra-aneurysmal flow diversion relative to the unstented ideal aneurysm model used as the control. However, the changes in flow pattern and velocity magnitude varied depending on the alterations in MCR caused by the discrepancy between the sizes of the PED and parent artery. The alteration of the direction of the inflow jet, which indicated flow diversion, was consistently observed in all aneurysm models implanted with PEDs, regardless of the variations in the sizes of the parent arteries. However, remnants of the distal-to-proximal jet flow were observed for the 3.0 mm configuration (1.0 mm oversizing). In contrast, the inflow jet was substantially reduced from the aneurysmal sac for the 4.5 mm configuration (undersized by 0.5 mm), which led to a distinct hemodynamic separation between the dome and neck of the aneurysm. Consequently, a higher MCR resulting from size matching or undersizing of PEDs led to decreased volume and velocity of the inflow jet, indicating an enhanced flow diversion effect.

### Reduction rate of hemodynamic parameters depending on the discrepancy between the sizes of the parent artery and flow diverter

The changes in the MCR, pore density, and hemodynamic parameters caused by the discrepancy between the sizes of the PED and parent artery are summarized in Table 1. The pore densities of the PED implanted in the neck of the aneurysm model with parent artery diameters of 3.0 mm (1.0 mm oversizing), 3.5 mm (0.5 mm oversizing), 4.0 mm (size matching), and 4.5 mm (0.5 mm undersizing) were found to be 28.5 pores/mm^2^, 31.9 pores/mm^2^, 45.5 pores/mm^2^, and 73.5 pores/mm^2^, respectively. This finding is consistent with the observed trend of the MCR, which increased during the transition from oversizing to undersizing.

**Table 1:** Reduction rates of the hemodynamic parameters according to the size of the parent artery.

Parent artery diameter (mm)	MCR (%)	Pore density (Pores/mm^2^)	Energy loss (W/m^3^) (Reduction rate %)	Avg. velocity (m/s) (Reduction rate %)	Inflow rate (mm^3^/s) (Reduction rate %)
Pipeline diameter (mm)					
Parent artery (3.0)	0	–	5235 (0.00)	0.0219 (0.00)	473.4 (0.00)
Pipeline (4.0)	25.5	28.5	2566 (50.99)	0.00923 (57.81)	202.4 (57.25)
Parent artery (3.5)	0	–	855.8 (0.00)	0.0169 (0.00)	285.7 (0.00)
Pipeline (4.0)	29.4	31.9	409.2 (52.19)	0.00330 (80.46)	81.98 (71.30)
Parent artery (4.0)	0	–	517.9 (0.00)	0.0116 (0.00)	232.9 (0.00)
Pipeline (4.0)	37.2	45.5	228.0 (55.98)	0.00192 (83.43)	54.82 (76.46)
Parent artery (4.5)	0	–	313.5 (0.00)	0.00859 (0.00)	167.1 (0.00)
Pipeline (4.0)	48.0	73.5	137.6 (56.12)	0.00137 (84.01)	38.40 (77.02)

*MCR* metal coverage rate.

**Figure 5:**
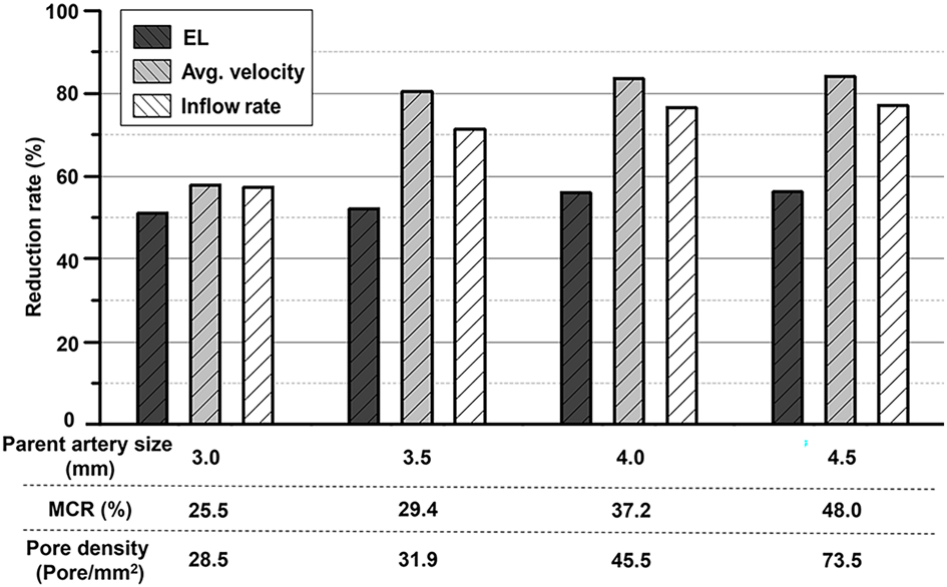
Reduction rates of the hemodynamic parameters depending on the size discrepancy between the flow diverter and parent artery. During the transition from oversizing to undersizing, as the metal coverage rate (MCR) and pore density increased, the reduction rate of the hemodynamic parameters also increased accordingly.

The PED implanted in a cerebral aneurysm model with a parent artery diameter of 4.5 mm (0.5 mm undersizing) showed the most significant reduction rates for all three hemodynamic parameters; the reduction rates were 56.12%, 84.01%, and 77.02% for the energy loss, average velocity, and inflow rate, respectively. On the other hand, the PED implanted in the cerebral aneurysm model with a parent artery diameter of 3.0 mm (1.0 mm oversizing) was associated with the least reduction rates for all three hemodynamic parameters; the reduction rates were 50.99%, 57.81%, and 57.25% for the energy loss, average velocity, and inflow rate, respectively. During the transition from oversizing to undersizing, the reduction rates of the hemodynamic parameters increased with increasing MCR and pore density (Table 1, Fig. 5).

Furthermore, the reduction rates of WSS at systole, TAWSS, and oscillatory shear index, have similar tendencies to that of the energy loss, average velocity, and inflow rate. (Supplementary Figure S2) When the PED was implanted in the cerebral aneurysm model with a parent artery diameter of 4.5 mm, the highest reduction rates were evaluated; the reduction rates were 86.02%, 83.96%, and 89.92% for the WSS at systole, TAWSS, and OSI. On the contrary, the lowest reduction rates were calculated in the cerebral aneurysm model with a parent vessel diameter of 3.0 mm; the reduction rates were 55.91%, 49.49%, and 58.33% for the WSS at systole, TAWSS, and OSI, respectively.

## Discussion

This study comprehensively analyzed the hemodynamic changes induced by the discrepancy between the sizes of the FD and parent artery in cerebral aneurysms, offering insights into the optimization of FD deployment for effective aneurysm management. Our findings demonstrate a correlation between MCR and flow diversion efficacy in aneurysm treatment influenced by the discrepancy between the sizes of the FD and parent artery. Oversizing the FD relative to the parent artery diameter significantly reduces the flow diversion performance. This reduction in hemodynamic performance with oversizing is evidenced by the decreased MCR and reduction rates of key hemodynamic parameters such as energy loss, average velocity, and inflow rate relative to those after undersizing.

A flow diverter is a type of braided stent with variable mesh densities attributed to its wire design. This flexibility is attributable to the adjustable wire configurations based on device size, vessel diameter, and curvature^21^. These adjustments result in significant porosity differences for cases using the same stent and along different parts of a single device^22^. Moreover, the relative size discrepancies between the device and parent artery critically influence the porosity and MCR, which affect the clinical performance of flow diverters^4,5,10^. Consistent with these findings, our study highlights that the discrepancies between the sizes of a flow diverter and the parent artery are important for determining changes in MCR (Fig. 3). Our study found that the MCR varied significantly with the size of the parent artery, increasing from oversizing to undersizing. The MCR ranged from 25.5% to 48.0%, with a clear trend towards higher values for cases with undersized FDs, for the aneurysm models with parent artery diameters ranging from 3.0 mm to 4.5 mm.

This study confirmed that the changes in MCR influenced the flow diversion effect via computational fluid dynamics analysis. As shown in Fig. 4, the changes in the flow pattern and velocity magnitude varied depending on the alterations in MCR caused by the discrepancy between the sizes of the PED and the parent artery. The observed variations in the flow patterns and velocity magnitudes underscore the advantages of size matching or undersizing over oversizing of flow diverters. Building on the insights of several previous qualitative studies, our findings highlight the influence of the size of an FD on the flow diversion effect based on the quantitative analysis of hemodynamic parameters using computational fluid dynamics. Kole et al.^7^ focused on the impact of the PED diameter on the treatment outcomes of intracranial aneurysms. They found that smaller-diameter PEDs, which have higher metal density, were more effective in occluding aneurysms. Furthermore, Mut et al.^6^ used patient-specific computational fluid dynamics models to assess the effects of FD oversizing on the cerebral aneurysm treatment outcomes. Their findings indicate that oversizing an FD can substantially reduce its efficacy in altering the intra-aneurysmal flow, highlighting the importance of appropriate sizing in clinical practice. All these insights emphasize the pivotal role of accurate sizing of FDs—matching or undersizing relative to the parent artery—in achieving optimal clinical results in aneurysm management through improved flow diversion effect.

This study quantitatively analyzed and compared the flow diversion effects based on the size discrepancy between the FD and the parent artery by examining changes in the reduction rates of hemodynamic parameters (Table 1, Fig. 5). The reduction rates of all hemodynamic parameters, including energy loss, average velocity, and inflow rate, increased with oversizing and vice versa with undersizing, demonstrating the superior flow diversion effect of undersizing. However, the study revealed that the increase in the reduction rates of hemodynamic parameters when parent artery diameters increased from 3.0 mm (oversizing) to 4.0 mm (matching) was not sustained as the diameters increased from 4.0 mm (matching) to 4.5 mm (undersizing). This finding suggests that undersizing the flow diverter can enhance the flow diversion effect but the benefits might plateau beyond a certain MCR. In addition, excessive undersizing in clinical practice may lead to complications, such as stent migration due to inadequate wall apposition^23^. Therefore, considering the findings of this study and the potential risk of complications associated with increased MCR^24,25^, appropriate sizing of the flow diverter is crucial to maximize outcomes by balancing risks and benefits.

This study has several limitations. First, the findings of this study were from experiments in controlled environments using idealized aneurysm models and stents to minimize bias and enable quantitative analysis. However, flow diversion performance is influenced by variable factors such as individual vascular geometry and the location and specifics of aneurysms in real-world practice. This underscores the need to consider patient differences and customized treatment strategies in deciding on the size of flow diverters. Second, the insufficient number of experiments conducted to firmly establish the reliability of the outcomes is another limitation. This study revealed a correlation between MCR and flow diversion effects using idealized stent and aneurysm models. However, various factors can affect MCR and flow diversion effectiveness, including deployment techniques such as degree of compaction, even when using the same stent and aneurysm model. Therefore, more experiments should be performed to increase the reliability of the results of this study. Third, the exclusive use of PEDs may have biased the results. All the experiments in this study were conducted solely with PEDs to control the bias from mechanical property differences among various FDs. This necessitates further research involving a wide range of commercially available FDs to understand how different devices lead to different outcomes. Despite these limitations, understanding the detailed hemodynamic alterations induced by the size discrepancy between the flow diverter and parent artery sizes identified this study will help clinicians can make more informed decisions in selecting appropriately sized flow diverters.

## Conclusions

The discrepancy between the sizes of the FD and parent artery in cerebral aneurysms significantly affects the MCR at the aneurysmal neck and the flow diversion effect. Oversizing of the flow diverter relative to the parent artery diameter markedly reduces flow diversion performance than undersizing. However, the flow diversion effect observed transitioning from oversizing to matching was less pronounced when moving from matching to undersizing. Understanding these hemodynamic impacts induced by size discrepancy, along with integrating diverse clinical considerations, will contribute to improved outcomes in aneurysm treatment by guiding the selection of appropriately sized FDs.

### Supplementary Information

Supplementary Figures.

## Data Availability

The datasets generated during and/or analysed during the current study are available from the corresponding author on reasonable request.

## References

[1] Lieber BB, Livescu V, Hopkins LN, Wakhloo AK (2002). Particle image velocimetry assessment of stent design influence on intra-aneurysmal flow. Ann. Biomed. Eng..

[2] Ohta M (2005). Rheological changes after stenting of a cerebral aneurysm: A finite element modeling approach. Cardiovasc. Intervent. Radiol..

[3] Xiang J (2014). Increasing flow diversion for cerebral aneurysm treatment using a single flow diverter. Neurosurgery.

[4] Makoyeva A, Bing F, Darsaut TE, Salazkin I, Raymond J (2013). The varying porosity of braided self-expanding stents and flow diverters: An experimental study. AJNR Am. J. Neuroradiol..

[5] Zhang M, Tupin S, Li Y, Ohta M (2021). Association between aneurysmal haemodynamics and device microstructural characteristics after flow-diversion treatments with dual stents of different sizes: A numerical study. Front. Physiol..

[6] Mut F, Cebral JR (2012). Effects of flow-diverting device oversizing on hemodynamics alteration in cerebral aneurysms. AJNR Am. J. Neuroradiol..

[7] Kole MJ (2019). Pipeline embolization device diameter is an important factor determining the efficacy of flow diversion treatment of small intracranial saccular aneurysms. J. Neurointerv. Surg..

[8] Zhang M (2017). Haemodynamic effects of stent diameter and compaction ratio on flow-diversion treatment of intracranial aneurysms: A numerical study of a successful and an unsuccessful case. J. Biomech..

[9] Kim Y (2023). 3D-printed patient-specific circles of willis with an intracranial aneurysm and their application to neurointerventional endovascular simulation. Adv. Mater. Technol..

[10] Shapiro M, Raz E, Becske T, Nelson PK (2014). Variable porosity of the pipeline embolization device in straight and curved vessels: A guide for optimal deployment strategy. AJNR Am. J. Neuroradiol..

[11] Dholakia R, Sadasivan C, Fiorella DJ, Woo HH, Lieber BB (2017). Hemodynamics of flow diverters. J. Biomech. Eng..

[12] Darsaut TE (2013). Flow diversion to treat aneurysms: The free segment of stent. J. Neurointerv. Surg..

[13] Tupin S, Saqr KM, Ohta M (2020). Effects of wall compliance on multiharmonic pulsatile flow in idealized cerebral aneurysm models: comparative PIV experiments. Exp. Fluids.

[14] Kim S, Yang H, Hong I, Oh JH, Kim YB (2021). Computational study of hemodynamic changes induced by overlapping and compacting of stents and flow diverter in cerebral aneurysms. Front. Neurol..

[15] Cho KC, Yang H, Kim JJ, Oh JH, Kim YB (2020). Prediction of rupture risk in cerebral aneurysms by comparing clinical cases with fluid-structure interaction analyses. Sci. Rep..

[16] Yang H (2022). Rupture risk prediction of cerebral aneurysms using a novel convolutional neural network-based deep learning model. J. Neurointerv. Surg..

[17] Yang H, Hong I, Kim YB, Cho K-C, Oh JH (2023). Influence of blood viscosity models and boundary conditions on the computation of hemodynamic parameters in cerebral aneurysms using computational fluid dynamics. Acta Neurochir. (Wien.).

[18] Takao H (2012). Hemodynamic differences between unruptured and ruptured intracranial aneurysms during observation. Stroke.

[19] Zhang M (2021). Implementation of computer simulation to assess flow diversion treatment outcomes: Systematic review and meta-analysis. J. Neurointerv. Surg..

[20] Li B (2022). Reliability of using generic flow conditions to quantify aneurysmal haemodynamics: A comparison against simulations incorporating boundary conditions measured in vivo. Comput. Methods Progr. Biomed..

[21] Damiano RJ (2017). Compacting a single flow diverter versus overlapping flow diverters for intracranial aneurysms: A computational study. AJNR Am. J. Neuroradiol..

[22] Darsaut TE, Bing F, Salazkin I, Gevry G, Raymond J (2012). Flow diverters can occlude aneurysms and preserve arterial branches: A new experimental model. AJNR Am. J. Neuroradiol..

[23] Al-Mufti F (2020). Bailout strategies and complications associated with the use of flow-diverting stents for treating intracranial aneurysms. Interv. Neurol..

[24] Berg P (2016). Endothelialization of over- and undersized flow-diverter stents at covered vessel side branches: An in vivo and in silico study. J. Biomech..

[25] Hassan T, Ahmed YM, Hassan AA (2011). The adverse effects of flow-diverter stent-like devices on the flow pattern of saccular intracranial aneurysm models: Computational fluid dynamics study. Acta Neurochir. (Wien.).

