# Supplementary material for: Quantitative analysis of hemodynamic changes induced by the discrepancy between the sizes of the flow diverter and parent artery
Source: Sci Rep. 2024 May 9;14:10653. doi: 10.1038/s41598-024-61312-y (PMC11081945; doi:10.1038/s41598-024-61312-y)
Supplement: Supplementary file 1 — Supplementary Figures. [file 41598_2024_61312_MOESM1_ESM.pdf]

Supplementary materials

Quantitative analysis of hemodynamic changes induced by the discrepancy between the sizes of the flow diverter and parent artery

Sunghan Kim, MD, PhD[1], Hyeondong Yang, PhD[2], Je Hoon Oh, PhD[2]*, Yong Bae Kim, MD, PhD[3]*

[1] Department of Neurosurgery, Bucheon St. Mary's Hospital, College of Medicine, The Catholic University of Korea, Seoul, Republic of Korea

[2] Department of Mechanical Engineering and BK21 FOUR ERICA-ACE Center, Hanyang University, Ansan, Gyeonggi-do, Korea

[3] Department of Neurosurgery, Severance Hospital, Yonsei University College of Medicine, Seoul, Korea

Sunghan Kim and Hyeondong Yang contributed equally to this work as first authors.

Je Hoon Oh and Yong Bae Kim contributed equally to this work as corresponding authors.

*Correspondence:

Je Hoon Oh, PhD.

Department of Mechanical Engineering and BK21 FOUR ERICA-ACE Center, Hanyang University, 55 Hanyangdaehak-ro, Sangnok-gu, Ansan, Gyeonggi-do 15588, Korea

Tel.: +82-31-400-5252

E-mail: jehoon@hanyang.ac.kr

Yong Bae Kim, MD, PhD.

Department of Neurosurgery, Severance Hospital, Yonsei University College of Medicine, 50-1 Yonsei-ro, Seodaemun-gu, Seoul 03722, Korea

Tel.: +82-2-313-5970

E-mail: ybkim69@yuhs.ac

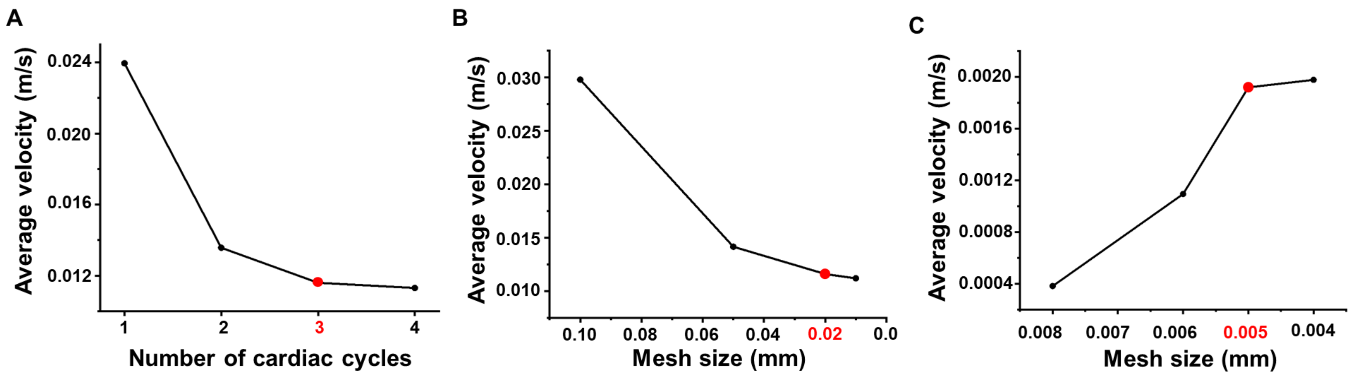

Supplementary Figure S1. Convergence test of the number of cardiac cycles (A) and mesh size for blood vessel (B) and flow diverter (C)

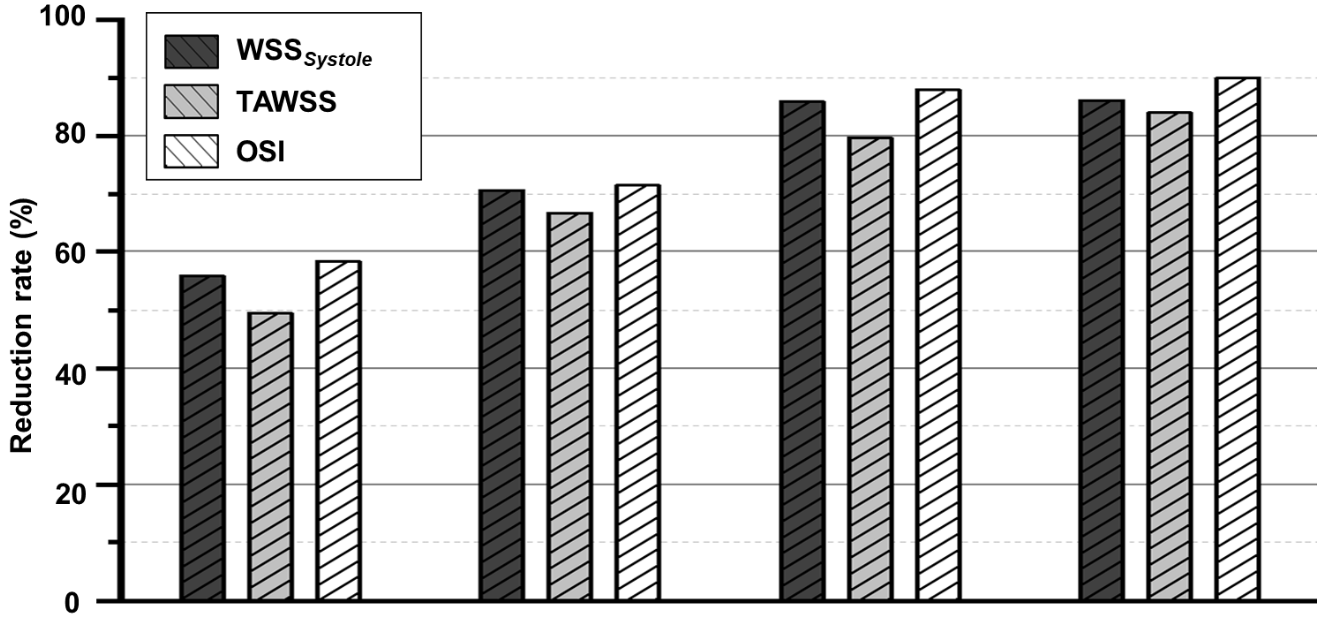

Parent artery size (mm)	3.0	3.5	4.0	4.5
MCR (%)	25.5	29.4	37.2	48.0
Pore density (Pores/mm^2)	28.5	31.9	45.5	73.5

Supplementary Figure S2. Reduction rates of the $WSS_{Systole}$, TAWSS, and OSI depending on the size discrepancy between the flow diverter and parent artery. WSS; wall shear stress, $WSS_{Systole}$; WSS at systole, TAWSS; time-average WSS, OSI; oscillatory shear index.
